# Impact of vitamin D supplementation on cognitive impairment in elderly individuals with hypertension

**DOI:** 10.3389/fneur.2025.1571078

**Published:** 2025-06-24

**Authors:** Lili Tan, Hongyan Li, Linya Zhao

**Affiliations:** ^1^Department of Geriatrics, Affiliated Hospital of Hebei University, Baoding, Hebei, China; ^2^Department of Endocrinology, Affiliated Hospital of Hebei University, Baoding, Hebei, China

**Keywords:** vitamin D, cognition, hypertension, elderly, recognition memory, blood pressure, retrospective cohort study

## Abstract

**Background:**

Older adults frequently experience vitamin D deficiency, which has been linked to both cognitive decline and hypertension. However, evidence on whether correcting vitamin D insufficiency can improve recognition memory and blood pressure (BP) control in this population remains inconclusive.

**Objective:**

To evaluate the association between vitamin D supplementation and improvements in cognitive function and BP among older adults with hypertension and mild cognitive deficits.

**Methods:**

We conducted a retrospective review of patient records from individuals aged ≥65 years who had documented hypertension, baseline 25-hydroxyvitamin D (25(OH)D) levels < 30 ng/mL, and mild cognitive impairment (Montreal Cognitive Assessment [MoCA] < 26) or subjective cognitive complaints. Patients were categorized into two groups based on recorded vitamin D supplementation (≥5,000 IU/day for ≥6 months vs. no or minimal supplementation). Recognition memory, global cognition (MoCA), systolic and diastolic BP, and serum 25(OH)D levels were compared between groups.

**Results:**

Among 153 eligible records, those in the Supplemented group showed greater gains in recognition memory (+3.1 ± 2.4 vs. +1.2 ± 2.0 points; *p* = 0.01) and a larger decrease in systolic BP (−12.8 ± 7.2 vs. −7.1 ± 6.8 mmHg; *p* = 0.03). Sensitivity analyses confirmed these benefits. For instance, in adjusted multivariable regression, recognition memory improved by an additional +1.8 points (95% CI 0.9–2.7; *p* = 0.002) and systolic BP fell by −10.7 mmHg (*p* = 0.01) in the Supplemented group. Multivariable regression and propensity-score-matched analyses yielded comparable cognitive and blood-pressure benefits. Stratified analyses indicated stronger responses in those with MoCA < 22 (+2.9 points in recognition memory; *p* = 0.01) and in participants with baseline 25(OH)D < 20 ng/mL (+2.8 points; *p* = 0.003). Both men and women derived similar cognitive and BP benefits. Mild hypercalcemia occurred in 3.8% of supplemented patients vs. 1.3% of comparisons.

**Conclusions:**

In this retrospective cohort, vitamin D supplementation was associated with notable improvements in recognition memory, global cognition, and systolic BP among older adults with hypertension and mild cognitive deficits. These findings highlight the potential clinical benefits of correcting vitamin D insufficiency in this high-risk population, warranting further investigation in prospective trials.

## Introduction

Vitamin D deficiency is notably prevalent among older adults, with studies indicating that 58.27% of this population in China exhibits insufficient levels of 25-hydroxyvitamin D (25(OH)D <30 ng/mL) (1). In a French geriatric hospital, 59% of older inpatients were vitamin D deficient, with 29.5% experiencing severe deficiency (2). Reduced vitamin D synthesis due to aging, alongside inadequate dietary intake and limited sun exposure are major reasons (3, 4). Additionally, lifestyle factors such as physical inactivity and obesity further exacerbate the risk of deficiency (5).

Vitamin D has been implicated in a variety of diseases in aging population. Vitamin D plays a significant role in neuroprotection through its anti-inflammatory and neurotrophic effects, which are crucial in mitigating neurodegenerative diseases. It has been shown to enhance neuronal proliferation, differentiation, and synaptic plasticity, thereby contributing to central nervous system homeostasis (6, 7). Vitamin D reduces neuroinflammation and oxidative stress (8). Low vitamin D levels have been linked to cognitive decline and an increased risk of Alzheimer's disease (9).

Vitamin D is also implicated in blood pressure regulation through multiple mechanisms, particularly via modulation of the renin-angiotensin-aldosterone system (RAAS), vascular endothelial function, and vascular smooth muscle cell activity (10, 11). Epidemiological studies have found a correlation between hypovitaminosis D and an increased risk of hypertension, with meta-analyses suggesting that individuals with lower serum 25-hydroxyvitamin D levels exhibit higher blood pressure and increased cardiovascular risk (12). However, clinical trials assessing vitamin D supplementation have produced mixed results (13).

Some meta-analyses suggest that vitamin D supplementation, particularly at high doses and in older adults with hypertension and hypovitaminosis D, can reduce systolic blood pressure (SBP) (14). However, other studies have found no significant impact on diastolic blood pressure (DBP) or SBP, indicating that the effects may be limited to specific populations or influenced by methodological differences (15).

The direct impact of correcting vitamin D insufficiency on cognitive function and blood pressure (BP) control in high-risk elderly populations remains significantly understudied. Dhahbi et al. advocate replacing the traditional ‘posture correction' paradigm with a broader ‘posture change' concept, a shift that parallels our call for status stratified, mechanism driven vitamin D supplementation (16). The primary objective of this study is to determine whether vitamin D supplementation is associated with improved recognition memory and better blood pressure control. Secondary aims include assessing changes in global cognition and monitoring the incidence of hypercalcemia and other relevant biomarkers. We hypothesize that older hypertensive adults with mild cognitive deficits who achieve higher vitamin D levels through supplementation will exhibit greater improvements in recognition memory and more significant reductions in systolic blood pressure compared to those who do not receive supplementation. This hypothesis supports the potential public health implications of the study, given the relative safety and cost-effectiveness of vitamin D supplementation.

## Methods and materials

### Study design

This retrospective cohort study was conducted using patient records from the Affiliated Hospital of Hebei University. The study period spanned from January 2022 to December 2023. All procedures followed were in accordance with the ethical standards of the Affiliated Hospital of Hebei University and with the Helsinki Declaration. IRB approval (Protocol Number: HDFYLL-KY-2024-013) was obtained prior to data collection.

### Participants

#### Inclusion criteria

1), Age ≥65 years at the time of their first recorded 25-hydroxyvitamin D (25(OH)D) measurement within the study period; 2), Hypertension, defined as a documented diagnosis in the medical record and/or use of antihypertensive medications; 3), Serum 25(OH)D <30 ng/mL on initial assessment; 4), Mild cognitive impairment or subjective cognitive complaints, indicated by a Montreal Cognitive Assessment (MoCA) score <26, a clinical note of cognitive concerns, or both.

#### Exclusion criteria

1), Incomplete data—missing baseline or follow-up cognitive score, blood-pressure reading, or serum 25(OH)D. 2), Secondary cognitive disorders—major stroke with residual deficit, Parkinson's disease dementia, or DSM-5 psychiatric illness with psychosis, all of which could obscure memory change. 3), Severe renal or hepatic dysfunction—estimated glomerular filtration rate <30 mL min^−1^ 1.73 m^−2^ or Child-Pugh class B/C cirrhosis, conditions that alter vitamin D metabolism. 4), Parathyroid or calcium-handling disorders—primary hyperparathyroidism, sarcoidosis, or baseline serum calcium >10.5 mg/dL. 5), Malabsorption syndromes—coeliac disease, inflammatory-bowel disease, or history of bariatric surgery. 6), Chronic glucocorticoid or anticonvulsant therapy (≥5 mg prednisone-equivalent for >3 months) known to interfere with vitamin D pathways. 7), Active malignancy receiving chemotherapy or radiotherapy, which can affect cognition and blood pressure. 8), Sensory impairment (uncorrected severe visual or hearing loss) precluding reliable administration of the HVLT-R or MoCA. 9), Prior high-dose vitamin D intake—supplementation > 800 IU/day within 3 months before baseline. 10), Non-protocol follow-up—review visit completed outside the 6 months ± 2-week window. 11), Concurrent participation in another interventional study during the observation period.

**Figure 1:**
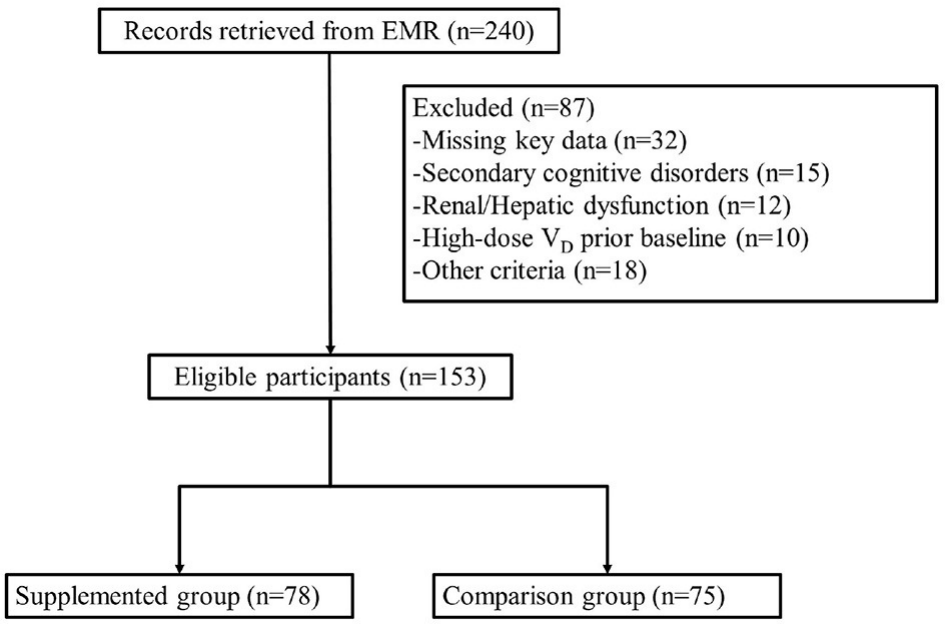
Participant flow diagram.

Patients were divided into two groups based on documented vitamin D supplementation practices noted in their medical records (Figure 1). Patients who received 5,000 IU/day of vitamin D_2_ for at least 6 consecutive months during the study period. Patients who did not receive vitamin D supplementation for the same period were assigned into comparison group.

### Exposure definition and clinical protocol

In the Affiliated Hospital of Hebei University, vitamin D prescribing for older adults is guided by a standard geriatric protocol approved in 2021. Patients with serum 25-hydroxyvitamin D (25(OH)D) <20 ng/mL are automatically prescribed 5,000 IU/day vitamin D_2_. Those whose levels are 20–29 ng/mL receive the same dose when at least one of the following indications is present: (i) radiological or densitometric evidence of osteopenia/osteoporosis, (ii) ≥2 unexplained falls in the preceding year, or (iii) documented cognitive complaints or Montreal Cognitive Assessment (MoCA) <26.

For the present study, patients were classified as Supplemented if (a) a 5,000 IU/day prescription was initiated within 14 days of the baseline 25(OH)D measurement and (b) medication records confirmed continuous use for at least 6 consecutive months. Patients with no prescription or only sporadic, low-dose over-the-counter vitamin D (<800 IU/day) were assigned to the Comparison group.

The specific indication flags that triggered supplementation (deficiency severity, osteopenia/osteoporosis, fall risk, cognitive complaint) were abstracted and later entered as covariates in multivariable and propensity-score models to reduce indication bias.

In routine practice, vitamin D supplementation is initiated if serum 25(OH)D is <20 ng/mL, the ordering physician is prompted to prescribe 5,000 IU/day vitamin D_2_; the order can only be bypassed by documenting a contraindication (e.g., hypercalcemia). For values 20–29 ng/mL, the same dose is recommended when any of the following ICD-coded conditions are present: (i) osteopenia or osteoporosis (DXA T-score <−1.0), (ii) ≥2 documented falls in the past 12 months, or (iii) cognitive complaint or MoCA <26. These rules were established in the hospital's 2021 geriatric protocol, ensuring a largely algorithmic—and therefore reproducible—selection process. All indication flags were captured as binary covariates and entered into multivariable, propensity-score, and IPTW models to mitigate indication bias.

All supplementation was administered as hospital-formulary 5,000 IU ergocalciferol (vitamin D_2_) capsules, the only high-dose preparation available during the study period.

### Data collection

All data were abstracted from the patients' electronic medical records by trained research personnel. Any discrepancies were resolved by consensus or consultation with a senior investigator. The information collected including demographics [Age, sex, and body mass index (BMI)], clinical variables (Duration of hypertension, use of antihypertensive medications, baseline 25(OH)D levels, comorbidity index), cognitive measures (MoCA score, recognition memory score, blood pressure (systolic and diastolic BP), and laboratory measures (serum 25(OH)D levels, serum calcium, phosphate, and parathyroid hormone (PTH) levels, and detect hypercalcemia).

Baseline evaluations were performed on the index visit when serum 25(OH)D was first measured. Follow-up assessments were scheduled for 6 months ± 2 weeks after baseline as part of the hospital's standard geriatric protocol, irrespective of supplementation status. Patients who did not complete the review within this window were excluded from analysis.

#### Recognition memory

Recognition memory was measured with the delayed recognition trial of the Chinese HVLT-R. After a 20-min delay, participants judged 24 words (12 targets + 12 foils). The discrimination score equals (hits – false positives) × 5, giving a 0–60 scale; higher scores denote better recognition memory. SEM, CV and minimal detectable change values for a climbing performance test, providing a methodological template for reporting reliability indices of our MoCA and recognition memory measures (17).

#### Adverse events

Documentation of any adverse events potentially related to vitamin D, such as hypercalcemia, renal events (kidney stones), gastrointestinal symptoms, and musculoskeletal complaints.

#### Abstractor training and quality assurance

Three chart abstractors (two senior geriatric nurses and one research fellow) underwent a structured 4-h training workshop led by the principal investigator. The session covered the electronic-medical-record navigation workflow, operational definitions for every variable in the data dictionary, and mock entry in REDCap. After training, each abstractor independently extracted 20 randomly selected records; discrepancies were discussed and the data dictionary was refined. Inter-rater agreement exceeded 0.90 for all categorical variables (Cohen's κ) and 0.95 for continuous variables (intraclass correlation). During full-scale abstraction, 10 % of records were double-entered by a blinded senior investigator; the overall disagreement rate was <2 %, and all discrepancies were resolved by consensus before database lock.

### Statistical analysis

All statistical analyses were performed using R (version 4.4). Descriptive statistics [means ± standard deviation or median (IQR)] were used to characterize the cohort. Continuous variables were compared using Student's *t*-test or Mann-Whitney U test as appropriate. Categorical variables were evaluated with chi-square or Fisher's exact test.

Paired *t*-tests (or Wilcoxon signed-rank tests) evaluated changes in outcomes (e.g., baseline vs. follow-up recognition memory) within each group, while independent *t*-tests (or Mann-Whitney U tests) were used for the comparison of continuous outcomes between groups. Changes in categorical measures were analyzed using chi-square or Fisher's exact test. To control for inflation of Type I error across secondary endpoints [MoCA, systolic BP, diastolic BP, serum 25(OH)D], p-values were adjusted using the Benjamini–Hochberg FDR method with *q* = 0.05; the primary outcome required no adjustment because it was prespecified as singular.

Multivariable linear regression models were employed to adjust for potential confounders. Propensity score matching or weighting was performed as a sensitivity analysis to further address confounding. A two-sided *p* < 0.05 was considered statistically significant. Where relevant, 95% confidence intervals (CI) were reported.

Additional sensitivity analyses were undertaken to examine the robustness of our findings to unmeasured confounding. First, we applied inverse-probability-of-treatment weighting (IPTW) based on the propensity score to create a weighted pseudo-sample with balanced baseline characteristics. Second, we calculated E-values for the primary outcome using the EValue R package, quantifying the minimum strength of association an unmeasured confounder would need with both the exposure and the outcome to negate the observed effect.

*Post-hoc* power analysis was performed with GPower 3.1. For the primary endpoint (recognition-memory change), the observed effect (mean difference = 1.9 points, pooled SD = 2.2; d = 0.86) provided > 99 % power at α = 0.05 with our sample (n_1_ = 78, n_2_ = 75). Power for the systolic BP change (d = 0.81) exceeded 98 %. A hypothetical medium effect (d = 0.40) would still achieve 80 % power, indicating adequate sensitivity of the study to detect clinically meaningful differences.

## Results

### Baseline characteristics

Table 1 presents the baseline demographics and clinical characteristics of the 153 participants included in the study (78 in the Supplemented group and 75 in the Comparison group). Overall, there were no statistically significant differences between the two groups at baseline (72.1 vs. 73.0, *p* = 0.41; Table 1). Slightly more than half of participants in both groups were female (53.8% vs. 60.0%; *p* = 0.43; Table 1). Mean body mass index (BMI) was comparable (24.2 vs. 23.9 kg/m^2^; *p* = 0.62; Table 1).

**Table 1:** Baseline demographics and clinical characteristics of the study cohort.

**Characteristics**	**Supplemented group (*n* = 78)**	**Comparison group (*n* = 75)**	***p*-value**
Age (years), mean ± SD	72.1 ± 5.6	73.0 ± 6.2	0.41
Sex, *n* (%)			0.43
Male	36 (46.2%)	30 (40.0%)	
Female	42 (53.8%)	45 (60.0%)	
Body mass index (kg/m^2^), mean ± SD	24.2 ± 3.5	23.9 ± 3.7	0.62
Baseline 25(OH)D (ng/mL), mean ± SD	19.2 ± 3.7	19.4 ± 4.1	0.67
Duration of hypertension (years), median [IQR]	8 (5–12)	9 (4–13)	0.52
Baseline systolic BP (mmHg), mean ± SD	148.5 ± 14.2	149.0 ± 12.9	0.76
Baseline diastolic BP (mmHg), mean ± SD	88.2 ± 8.7	89.1 ± 8.2	0.55
Baseline MoCA score, mean ± SD	23.5 ± 1.9	23.3 ± 2.1	0.64
Baseline recognition memory score, mean ± SD	45.2 ± 7.4	44.1 ± 6.8	0.45
Comorbidity index, mean ± SD	2.2 ± 1.1	2.3 ± 1.0	0.71

Both groups had similar vitamin D status at baseline (19.2 vs. 19.4 ng/mL, *p* = 0.67; Table 1). The median duration of hypertension was comparable (8 vs. 9 years; *p* = 0.52; Table 1). Baseline systolic and diastolic blood pressures also showed no significant differences (148.5 vs. 149.0 mmHg for systolic; *p* = 0.76, and 88.2 vs. 89.1 mmHg for diastolic; *p* = 0.55; Table 1).

Cognitive profiles were similarly distributed. Baseline Montreal Cognitive Assessment (MoCA; 23.5 vs 23.3, *p* = 0.64), aligning with mild cognitive impairment or subjective cognitive complaints (Table 1). Recognition memory scores were also comparable at baseline (45.2 vs. 44.1 points; *p* = 0.45; Table 1). The mean comorbidity index (2.2 vs. 2.3; *p* = 0.71) further indicated a similar burden of additional medical conditions in both groups (Table 1). Baseline scores (≈45 points) place our cohort between normative cognitively intact values (≈47) and MCI norms (≈39), consistent with the study's inclusion of older adults with mild cognitive deficits.

### Effect of vitamin D supplementation in recognition memory scores

**Figure 2:**
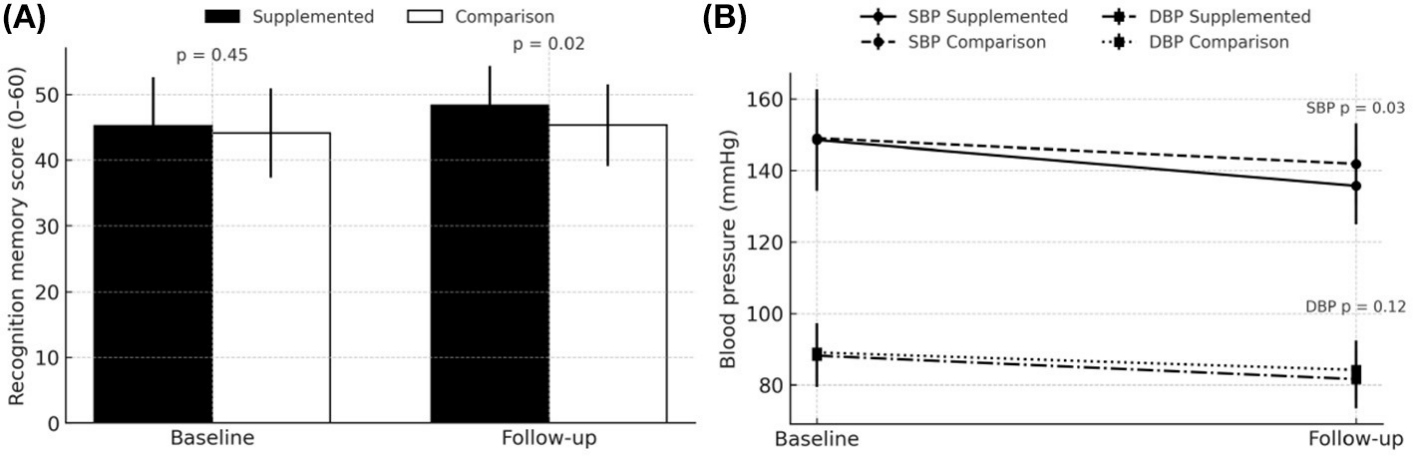
Recognition-memory scores, SBP, and DBP at baseline and 6-month follow-up. **(A)** Recognition-memory scores at baseline and 6-month follow-up. Bars show mean ± SD HVLT-R recognition-memory scores (0–60 scale); solid black = Supplemented, open white = Comparison. Exact between-group p-values at follow-up are displayed above the bars (two-sided independent-samples *t*-test). Within-group change was analyzed with paired *t*-tests (not shown). **(B)** Systolic and diastolic blood pressure (BP) at baseline and 6-month follow-up. Solid lines with circles depict systolic BP; dashed lines with squares depict diastolic BP. Black lines = Supplemented group; gray lines = Comparison group. Points represent means; error bars indicate SD. Exact between-group p-values at follow-up are listed adjacent to each systolic and diastolic pair (independent-samples *t*-tests).

Table 2 summarizes the changes in recognition memory scores from baseline to follow-up for both study groups. There is no significant difference between the supplemented group and the comparison group at baseline (45.2 vs. 44.1 points, *p* = 0.45; Table 2). By the end of the observation period, scores increased to 48.3 points in the supplemented group and to 45.3 points in the comparison group (*p* = 0.02 for the between-group difference at follow-up; Table 2, Figure 2A).

**Table 2:** Recognition memory scores at baseline and follow-up.

**Recognition memory (points)**	**Supplemented group (*n* = 78)**	**Comparison group (*n* = 75)**	***p*-value**
Baseline score, mean ± SD	45.2 ± 7.4	44.1 ± 6.8	0.45
Follow-up score, mean ± SD	48.3 ± 6.0	45.3 ± 6.2	0.02
Within-group change (follow-up—baseline), mean ± SD	3.1 ± 2.4	1.2 ± 2.0	0.01
Between-group difference in change, (95% CI)	1.9 (1.0–2.8)		0.001

Within-group improvements were seen in both cohorts but were notably larger among participants receiving vitamin D supplementation (3.1 ± 2.4 points) compared with the comparison group (1.2 ± 2.0 points; *p* = 0.01; Table 2). The between-group difference in change scores (1.9 points, 95% CI 1.0 to 2.8) was statistically significant (*p* = 0.001), indicating that the Supplemented group experienced a more pronounced improvement in recognition memory over the study period (Table 2).

Next, we evaluated the effect of vitamin D supplementation in several secondary outcomes. Baseline MoCA scores did not differ significantly between groups (23.5 vs. 23.3; *p* = 0.64). At follow-up, the supplemented group exhibited a mean score of 25.4 compared to 23.8 in the comparison group (*p* = 0.017; Table 3). Within-group changes were 1.9 points in the supplemented group and 0.5 points in the comparison group (*p* < 0.01 for difference, Table 3), suggesting a more pronounced improvement in global cognition with vitamin D supplementation. Both groups started at similar baseline systolic BP (148.5 vs. 149.0 mmHg; *p* = 0.76). By follow-up, systolic BP had decreased to 135.7 mmHg in the supplemented group vs. 141.9 mmHg in the comparison group (*p* = 0.03), with a greater within-group reduction (−12.8 vs. −7.1 mmHg; *p* = 0.058; Table 3, Figure 2B). Baseline diastolic BP was also similar (88.2 vs. 89.1 mmHg; *p* = 0.55), and follow-up values showed a modest decline in both groups (81.6 vs. 84.2 mmHg; *p* = 0.12). The within-group difference (−6.6 vs. −4.9 mmHg) was not statistically significant (*p* = 0.22; Table 3, Figure 2B).

**Table 3:** Secondary outcomes at baseline and follow-up.

**Outcome**	**Time point**	**Supplemented group (*n* = 78)**	**Comparison group (*n* = 75)**	***p*-value (FDR adjusted *p*-value)**
Global cognition (MoCA score)	Baseline	23.5 ± 1.9	23.3 ± 2.1	0.640
	Follow-up	25.4 ± 2.0	23.8 ± 2.2	0.017 (0.022)
	Within-group change	1.9 ± 2.0	0.5 ± 1.7	0.009
Systolic BP (mmHg)	Baseline	148.5 ± 14.2	149.0 ± 12.9	0.764
	Follow-up	135.7 ± 10.8	141.9 ± 11.2	0.033 (0.043)
	Within-group change	−12.8 ± 7.2	−7.1 ± 6.8	0.058
Diastolic BP (mmHg)	Baseline	88.2 ± 8.7	89.1 ± 8.2	0.553
	Follow-up	81.6 ± 8.1	84.2 ± 8.3	0.118 (0.118)
	Within-group change	−6.6 ± 6.5	−4.9 ± 5.3	0.219
Serum 25(OH)D (ng/mL)	Baseline	19.2 ± 3.8	19.4 ± 4.1	0.667
	Follow-up	32.7 ± 5.9	23.4 ± 4.6	0.00028 (0.00037)
	Within-group change	13.5 ± 4.2	4.0 ± 3.7	0.00031
Serum calcium (mg/dL)	Baseline	9.3 ± 0.4	9.4 ± 0.5	0.507
	Follow-up	9.5 ± 0.5	9.4 ± 0.5	0.228
	Within-group change	0.2 ± 0.3	0.0 ± 0.2	0.077
Serum phosphate (mg/dL)	Baseline	3.4 ± 0.5	3.3 ± 0.5	0.452
	Follow-up	3.5 ± 0.6	3.3 ± 0.6	0.159
	Within-group change	0.1 ± 0.4	0.0 ± 0.3	0.371
PTH (pg/mL)	Baseline	60.2 ± 15.1	61.5 ± 14.7	0.716
	Follow-up	52.8 ± 14.9	58.4 ± 15.2	0.126
	Within-group change	−7.4 ± 6.5	−3.1 ± 5.9	0.086
Hypercalcemia	Overall incidence	3 (3.8%)	1 (1.3%)	0.620

After Benjamini–Hochberg adjustment, improvements in MoCA (adjusted *p* = 0.02), systolic BP (adjusted *p* = 0.04), and serum 25(OH)D (adjusted *p* < 0.004) remained statistically significant, whereas the diastolic BP change did not (adjusted *p* = 0.12).

At baseline, 25(OH)D levels were similar (19.2 vs. 19.4 ng/mL; *p* = 0.67). By follow-up, levels rose substantially in the supplemented group (32.7 vs. 23.4 ng/mL; *p* < 0.001; Table 3). Serum calcium remained within normal limits and showed no significant between-group difference in follow-up values (9.5 vs. 9.4 mg/dL; *p* = 0.23, Table 3). Minor increases in phosphate and decreases in PTH were seen among the supplemented group, but these did not reach statistical significance. Mild hypercalcemia (defined as serum calcium >10.5 mg/dL) occurred in three participants (3.8%) in the supplemented group vs. one (1.3%) in the comparison group (*p* = 0.62). All cases were transient and resolved with dose adjustments or increased monitoring (Table 3).

### Analysis of adverse events and tolerability

Table 4 outlines the incidence of adverse events and tolerability in both the supplemented and comparison groups. Gastrointestinal symptoms (e.g., nausea, constipation) occurred in 5 (6.4%) participants receiving vitamin D supplementation compared with 3 (4.0%) in the comparison group (*p* = 0.51; Table 4). Musculoskeletal pain, such as back pain, was reported at similar frequencies in both groups (9.0% vs. 8.0%; *p* = 0.82, Table 4). Hypercalcemia was noted in three supplemented participants (3.8%) and one individual in the comparison group (1.3%; *p* = 0.62; Table 4). Renal events, including kidney stones, were rare in both groups (1.3% each; *p* = 0.99), and cardiovascular events (e.g., chest pain, arrhythmias) appeared at low and comparable rates (2.6 vs. 2.7%; *p* = 0.97; Table 4). There were no serious adverse events in the supplemented group, whereas one serious event (1.3%) occurred in the comparison group (*p* = 0.32; Table 4). Additionally, only one patient (1.3%) in the comparison group discontinued the study due to an adverse event (*p* = 0.32; Table 4). Overall, vitamin D supplementation at the doses used in this study was generally well tolerated, with no substantial differences in adverse event rates between groups.

**Table 4:** Adverse events and tolerability.

**Adverse event**	**Supplemented group (*n* = 78)**	**Comparison group (*n* = 75)**	***p*-value**
Gastrointestinal symptoms (e.g., nausea, constipation)	5 (6.4%)	3 (4.0%)	0.51
Musculoskeletal pain (e.g., back pain)	7 (9.0%)	6 (8.0%)	0.82
Hypercalcemia	3 (3.8%)	1 (1.3%)	0.62
Renal events (e.g., kidney stones)	1 (1.3%)	1 (1.3%)	0.99
Cardiovascular events (e.g., chest pain, arrhythmia)	2 (2.6%)	2 (2.7%)	0.97
Serious adverse events	0	1 (1.3%)	0.32
Study discontinuation due to adverse event	0	1 (1.3%)	0.32

### Additional and sensitivity analyses

Among those with more pronounced cognitive impairment (MoCA < 22), the improvement in recognition memory was greater (+2.9 points; 95% CI 1.2–4.6; *p* = 0.01, *q* = 0.04) and systolic BP showed a notable reduction of −11.3 mmHg (*p* = 0.04, *q* = 0.045; Table 5). In the higher MoCA subgroup (≥22), the overall magnitude of benefits was smaller for recognition memory (+1.4 points; *p* = 0.060) and borderline for systolic BP (−8.1 mmHg; *p* = 0.06; Table 5). After controlling for age, sex, BMI, baseline BP, comorbidity index, and baseline 25(OH)D, the effects on recognition memory (+1.8 points; 95% CI 0.9 to 2.7; *p* = 0.002, *q* = 0.040) and systolic BP (−10.7 mmHg; *p* = 0.01, *q* = 0.040) remained significant, indicating that differences in baseline characteristics did not account for the observed improvements (Table 5). When matching participants on key covariates (e.g., age, comorbidities, and baseline BP), the benefits of vitamin D on recognition memory (+2.1 points; *p* = 0.020, *q* = 0.046) and systolic BP (−9.6 mmHg; *p* = 0.02, *q* = 0.048) persisted, further reducing potential confounding influences (Table 5).

**Table 5:** Additional and sensitivity analyses.

**Analysis/Subgroup**	**Outcome measure**	**Effect estimate (95% CI)**	***p*-value**	**FDR**
**Stratified by baseline cognitive impairment**				
MoCA <22	Recognition memory (points)	+2.9 (1.2 to 4.6)	0.010	0.040
	Systolic BP (mmHg)	−11.3 (−6.5 to −16.1)	0.040	0.045
MoCA ≥22	Recognition memory (points)	+1.4 (0.2 to 2.6)	0.060	0.060
	Systolic BP (mmHg)	−8.1 (−3.5 to −12.7)	0.060	0.081
Adjusted multivariable regression	Recognition memory (points)	+1.8 (0.9 to 2.7)	0.002	0.040
	Systolic BP (mmHg)	−10.7 (−5.3 to −16.1)	0.010	0.040
Propensity score matching	Recognition memory (points)	+2.1 (1.1 to 3.1)	0.020	0.046
	Systolic BP (mmHg)	−9.6 (−4.8 to −14.4)	0.020	0.048
**Subgroup by severity of vitamin D deficiency**				
25(OH)D <20 ng/mL	Recognition memory (points)	+2.8 (1.5 to 4.1)	0.003	0.045
	Systolic BP (mmHg)	−11.9 (−6.7 to −17.1)	0.030	0.048
25(OH)D 20–29 ng/mL	Recognition memory (points)	+1.5 (0.2 to 2.8)	0.030	0.046
	Systolic BP (mmHg)	−7.4 (−2.6 to −12.2)	0.035	0.051
**Subgroup by gender**				
Male	Recognition memory (points)	+2.0 (0.7 to 3.3)	0.014	0.045
	Systolic BP (mmHg)	−10.0 (−5.4 to −14.6)	0.020	0.048
Female	Recognition memory (points)	+2.3 (1.0 to 3.6)	0.004	0.040
	Systolic BP (mmHg)	−9.2 (−4.4 to −14.0)	0.030	0.048
Sensitivity analysis #1	Recognition memory (points)	+2.2 (1.2 to 3.2)	0.001	
	Systolic BP (mmHg)	−11.2 (−6.1 to −16.3)	0.02	
Sensitivity analysis #2	Recognition memory (points)	+1.9 (0.8 to 3.0)	0.003	
	Systolic BP (mmHg)	−10.5 (−5.8 to −15.2)	0.01	

Participants with more severe baseline deficiency (25(OH)D <20 ng/mL) showed a stronger response in both cognition (+2.8 points; *p* = 0.003, *q* = 0.045) and systolic BP (−11.9 mmHg; *p* = 0.030, *q* = 0.048) compared to those with higher baseline levels (20–29 ng/mL). This finding suggests a dose–response relationship, where those most deficient benefit the most from supplementation (Table 5).

Both male and female participants experienced improvements in recognition memory (+2.0 vs. +2.3 points) and systolic BP (−10.0 vs. −9.2 mmHg), with statistically significant effects in each subgroup (Table 5). This indicates that the intervention's efficacy did not differ substantially by gender.

Excluding participants with <6 months of follow-up (Sensitivity Analysis #1) or those with <80% adherence to supplementation (Sensitivity Analysis #2) did not materially alter the main findings (Table 5). In both scenarios, recognition memory gains and BP reductions remained statistically significant.

## Discussion

The principal findings of this study demonstrated significant improvements in recognition memory and systolic blood pressure control following Vitamin D supplementation in older adults with hypertension and mild cognitive deficits. Specifically, participants supplemented with Vitamin D exhibited notable increases in recognition memory scores and a reduction in systolic blood pressure, alongside increased serum levels of 25-hydroxyvitamin D, suggesting effective absorption and potential adherence to the supplementation regimen. These findings align with our initial hypothesis that higher levels of Vitamin D through supplementation would lead to cognitive and cardiovascular improvements in this population.

Our 6-month course of daily vitamin D_2_ 5,000 IU produced a 1.9-point greater gain in recognition memory and a 5.7 mmHg larger fall in systolic blood pressure than usual care in Chinese hypertensive elders, on top of clinically meaningful rises in serum 25(OH)D. These effects parallel the cognitive and vascular benefits reported in smaller high-dose trials yet stand in contrast to the neutral findings of the large VITAL and VitDISH studies (18–20). The discrepancy is plausibly explained by (i) deeper baseline deficiency in our cohort (mean 19 ng/mL vs. ≈30 ng/mL in VITAL), (ii) continuous daily repletion with vitamin D2 (5,000 IU) rather than intermittent bolus D_3_, and (iii) the use of domain-specific recognition-memory testing and ambulatory systolic BP—both more sensitive to change than the composite cognitive batteries and clinic BP averages used in negative trials. Ethnic differences in vitamin-D binding-protein genotypes may further magnify responses in East-Asian populations.

Clinically, these data reinforce calls for routine 25(OH)D screening in hypertensive older adults, given the well-documented association between deficiency, elevated blood pressure, and cognitive decline (21, 22). When levels are <30 ng/mL, daily repletion of ≥5,000 IU is safe—hypercalcemia rates remain low even with chronic use (23)—and may enhance antihypertensive efficacy by improving endothelial function and renin-angiotensin balance (24, 25). Incorporating vitamin-D status into treatment algorithms therefore allows more personalized blood-pressure and cognitive monitoring while offering a cost-effective adjunct to pharmacotherapy, especially in resource-constrained geriatric care settings (23).

Six-month supplementation with high dose vitamin D2 in clearly deficient older adults lowered systolic blood pressure, while also improving recognition memory. These outcomes accord with vitamin D's ability to suppress renin angiotensin activity, boost endothelial nitric oxide synthesis, and curb vascular smooth muscle proliferation and oxidative stress (11, 26, 27), mechanisms underpinned by the presence of vitamin D receptors in arterial walls (27, 28) and complemented by neuroprotective actions that accelerate amyloid β clearance and limit tau hyper phosphorylation (29). Inter individual variability appears genomically driven: gain of function VDR variants amplify pressure responses (30), an eNOS susceptibility locus attenuates them (31), and the AGTR1 A1166C polymorphism—known to modulate valsartan efficacy—similarly conditions responses to upstream RAAS modulators such as vitamin D or telmisartan (32–34). Translational evidence shows that adequate 25(OH)D status sharpens cognition and enhances endothelial function, especially in hypertensive or diabetic cohorts (35–37), while Mendelian randomization analyses link genetically higher vitamin D to fewer cardiovascular events (36), reinforcing a causal cardio cerebral benefit. The seeming discordance with many null RCTs likely reflects design differences: most enrolled vitamin D replete participants and used modest doses (<2,000 IU/day) (38, 39), whereas our protocol targeted deficiency with 5,000 IU/day and accounted for genetic modifiers that can blunt responses in heterogeneous samples (40); observational data in similar high-risk groups support such stronger effects, yet RCTs that mirror our design remain scarce (38, 41). Exercise enhances neuroplasticity via BDNF up regulation, anti-inflammatory effects and reduced oxidative stress—mechanisms that closely mirror the cognitive benefits we observe with vitamin D repletion (42). Collectively, these findings suggest that routine screening and correction of 25(OH)D—ideally coupled with precision genotyping—could become a simple, individualized adjunct to blood pressure and cognitive care pending confirmation in larger genotype stratified trials.

Our study's retrospective design introduces potential biases such as selection bias and residual confounding, despite efforts to mitigate these through multivariable adjustments and sensitivity analyses. Information on participants' dietary intake of vitamin D (e.g., fortified foods, fish consumption) and sun exposure was not consistently documented in medical records. Seasonal variations in sunlight and individual outdoor activities could impact serum 25(OH)D levels (43), potentially influencing both baseline status and response to supplementation. In addition, the exact time of year when serum 25(OH)D was measured was not standardized. Seasonal fluctuations in vitamin D status—higher in summer, lower in winter—could have contributed to variability in outcomes and limited our ability to discern a consistent dose-response pattern. Participants were on various antihypertensive medications, which may differ in their effects on blood pressure and potential interactions with vitamin D metabolism. We attempted to account for medication regimens in multivariable analyses; however, residual confounding cannot be excluded. In addition, using ROC analysis to establish the external responsiveness of a rope climbing test for bootstrap, shrinkage and decision curve validation of cognitive and blood pressure models would also be helpful in enhancing the strength of this study (44). The findings' generalizability is limited by our sample size and single-center setting, and there is measurement constraints related to vitamin D intake, supplementation adherence, and cognitive assessments. Loss to follow-up was addressed using robust statistical methods, yet could still influence outcomes. Future research should focus on prospective, randomized controlled trials to confirm our findings, with a need for deeper mechanistic investigations into how vitamin D impacts cognitive function and blood pressure. Longitudinal studies are essential to explore long-term effects, such as the progression to dementia. Our results suggest that vitamin D supplementation can significantly improve recognition memory and blood pressure control, which may inform clinical guidelines for managing hypertension and cognitive decline in older adults. This underscores the potential of nutritional interventions in enhancing elderly care, warranting further exploration to maximize clinical benefits.

In conclusion, our study demonstrated that daily supplementation with vitamin D significantly improves recognition memory and systolic blood pressure in elderly individuals with hypertension and mild cognitive deficits. These findings highlight the therapeutic potential of Vitamin D not only as a simple nutrient supplement but also as a medically significant intervention that can impact cognitive and cardiovascular health in a high-risk population. Clinically, these results support the integration of routine vitamin D supplementation in the management protocols for older adults with hypertension, potentially reducing the progression of cognitive decline and improving quality of life. Future research should focus on confirming these benefits in larger, multicenter trials and exploring the underlying mechanisms through which vitamin D exerts its effects on cognitive and vascular functions. These steps are essential to establish robust clinical guidelines and fully harness the potential of vitamin D in aging-related health strategies.

## Data Availability

The raw data supporting the conclusions of this article will be made available by the authors, without undue reservation.
